# Abrégé Pratique des Maladies de la Peau, d'après les Leçons de Biett

**Published:** 1832-04-01

**Authors:** 


**ABRÉGÉ PRATIQUE DES MALADIES DE LA PEAU, D'APRES LES AUTEURS LES PLUS ESTIMÉS, ET SURTOUT D'APRES DES DOCUMENS PUISÉS DANS LES LEÇONS CLINIQUE DE M. LE DOCTEUR BIETT, MÉDECIN DE L'HÔPITAL ST. LOUIS. Par *Alphée Cazenave*, et *H. E. Schedel*, Docteurs en Médecine, Anciens Internes de l'Hôpital St. Louis, &c. A Paris, chez Béchet, Jeune, 1828.**

THE practical object of classification in diseases, is, by their judicious assortment, and lucid arrangement into small groups, to facilitate their study, and by one common nomenclature founded on accurate and definite descriptions, to enable all to understand more intelligibly the same affection by the same term;—its more philosophic design is to shew the affinities of the several diseases amongst themselves, and of the groups with each other, in order to lay down general rules for treatment, and to endeavour (as in natural science) to discover the general laws which influence morbid action. That classification can be alone correct which results from the careful observation of the symptoms of each disease during its whole course, the accurate examination of the changes discovered after death, the comparison of these among themselves, and the grouping together of those diseases which in all material points agree with each other. The proximate causes of diseases are so little understood that it is dangerous to build even a part of a classification on any theoretic explanation of them; and on looking back to the various nosologies that have been produced, this reliance on theories rather than on facts, together with deficient pathological knowledge, will be found to have been fruitful sources of error. The Nosologia Methodica of Sauvages, published in 1762, was the first attempt of any magnitude at the general arrangement of diseases;—it was a laborious production, the result of great personal experience as well as extensive reading. If morbid anatomy had then been cultivated with the zeal and success which has attended its more extended and useful cultivation of late years, Sauvages would hardly have placed hydrothorax in his class "Anhelationes," and separated ophthalmia from the phlegmasiæ, placing it in the 7th class "Dolores." If Cullen had not left the safe path of accurate observation, and bent his facts to his peculiar theories, he would hardly have placed diarrhœa and diabetes under spasmi. Some physicians have objected to nosologies, but their objections can alone apply to those founded on theories. The advantages of a classification, the result of close and accurate observation, are exhibited in a strong light in the system of cutaneous diseases by Willan. Previously to the publication of his work no diseases were surrounded with so much obscurity and less understood; which was the more surprising from their affording better opportunities of investigation than any other classes of morbid alterations, as both the senses of vision and touch can be immediately brought to bear on their elucidation. The different significations attached to the word "lepra" are examples of the obscurity and confusion arising from the loose and inaccurate definitions of the primary forms of eruptions which were the great obstacles to the attainment of any clear knowledge of cutaneous diseases: thus, it has been applied to a tuberculous eruption, the elephantiasis of the Greeks; to a chronic enlargement of the subcutaneous cellular tissue, the elephantiasis of the Arabs; to a circular squamous disease, the lepra vulgaris; and to a union of many eruptions, as eczema, psoriasis, and lichen. Willan founded his system on the primary forms of cutaneous diseases, and commenced by giving a precise definition of the forms he applied to them. His eight orders of Papulæ, Squamæ, Exanthemata, Bullæ, Vesiculæ, Pustulæ, Tubercula, and Maculæ, were so named from distinct elementary lesions, and subsequent experience has proved that, although some alterations can be beneficially made in the details, the general plan is, both as it regards facility of study and precision, the best that can be adopted. Alibert in France brought out his classification eight years subsequently to that of Willan; it was founded on that of "mercuriali," in a work "De Morbis Cutaneis," &c. published at Venice in 1572. Alibert commences by dividing, first of all, cutaneous diseases into two groups, those of the head, ("teignes,") and those of the body, ("dartres,")—he subdivides these into species and varieties: thus, if there is a scaly eruption on the body, he names it "dartre squammeuse;" if it is attended with a serous exhalation, or is of a circular form, he adds to "dartre squammeuse" either the term "humide" or "orbiculare," indicating the variety. Besides five species of "teignes" and seven "dartres," he separately describes "pliques, ephelides, cancroides, leprés, pians, icthyoses, syphilides, scrophules, psorides. Thus it has not the advantage of simplicity. The first divisions into eruptions affecting the skin of the head and those of the body is incorrect, as there is no species of eruption which has so special a seat that it can never be met with in other regions of the body with analogous characters; another disadvantage is, that of giving the same name to eruptions differing entirely in their primary form, in their progress, and in the treatment they require, and which are only similar at a particular stage of the disease when superficially examined. Thus M. Alibert comprehends under "dartre squammeuse humide," both some forms of eczema, a clearly vesicular affection, attended with the discharge of a fluid which forms yellowish scabs, and lichen agrius, a papular eruption which is attended with so much inflammation and painful tension, that in a later stage of the disease the summits of the papula ulcerate, and scabs are formed: now, in the first stage of lichen agrius it is neither "squammeuse" nor "humide," and must therefore be placed in another section. This classification, although almost unknown in this country, has had many disciples in Germany as well as in France. This was owing, in great measure, perhaps, to the magnificent and attractive folio work which M. Alibert has published, (and of which Dr. Bateman has rather too severely said, "that the merit of his publication belongs principally to the artists which he has had the good fortune to employ,") and to the opportunities the author of it has had of promulgating his opinions in his popular course of lectures delivered at the Hôpital St. Louis, for many years without a rival; the apparent simplicity of the great primary division into two classes was a source of considerable attraction, libenter amplectuntur ea præcepta quæ sedulitatem non exigunt; and the general terms "dartre" and "teigne," although conveying no distinct meaning, were most readily applied, and afforded a convenient cloak for ignorance. Eleven years since M. Biett, a co-physician of M. Alibert's]

at the Hôpital St. Louis, commenced a course of clinical lectures on cutaneous diseases, founded on the system of Willan and Bateman, which he slightly modified in a few non-essential details. Perhaps to those who may be unacquainted with the system of Paris hospitals, it will be as well to inform them that, unlike similar institutions in our own country, they are not the offspring of private munificence, but are wholly provided for by the government, which appoints a committee for their general superintendence: a tax which is taken at the barriers of Paris is applied to their maintenance. All invalids (except those who from severe accidents require immediate admission) first apply at a central office, where, after an examination, they are distributed to the various hospitals, but not indiscriminately, as some of these are set apart for peculiar diseases; thus the syphilitic patients are sent to the Hôpital des Venérins, children to the Hôpital des Infans, and those affected with cutaneous eruptions to the Hôpital St. Louis. This last hospital is of considerable extent, and Messrs. Alibert and Biett have each the care of nearly 100 beds, containing patients affected with skin diseases alone: as the out-patients are still more numerous, few institutions in the world can afford better opportunities for the study of a particular subject. To these advantages M. Biett adds that of a large private practice, the engagements of which, as well as a dislike, quite unusual among his countrymen, of publishing, are the causes of no work on a subject to which he has devoted his time, and powers of observation, having as yet proceeded from his pen. He has therefore found that his peculiar opinions have been borrowed from his lectures by many authors in a most wholesale manner, without acknowledgment. This was particularly the case in a work on skin diseases, published by a young physician of La Charité, named Rayer. Consequently two of M. Biett's former "internes," or house-surgeons, who had been in his wards several years, have brought out this work founded principally on his clinical observations and lectures, and therefore containing his opinions and modes of treatment. This volume is compiled throughout with that system which marks the later publications of the French medical press; the symptoms, causes, diagnosis, prognosis, and treatment, are given under separate heads in the description of each genus; and although the species and varieties do not agree "in toto" with those of Bateman, as they are less numerous, yet the names given by him, as well as by Alibert, are introduced where the form of the diseaee to which they have been applied is described. It will be the object of this review not to copy descriptions which have previously been accurately made by Willan and perfected by Bateman, in his admirable "Synopsis," but to point out the alterations and improvements in classification, description, and treatment which M. Biett has deduced from the most extensive experimental research. Numerous remedies which hitherto have been but little employed in this country, have had in his hospital a trial on a large scale, such as the iodurets of mercury, sulphur, &c. and it will be allowed that the efficacy of medicated and other baths may be estimated, when it is stated that 150,000. are given annually at the Hôpital St. Louis.

The eight orders of Willan are taken, but, instead of including all skin diseases under these heads, seven genera are omitted, as of an anomalous nature—these are lupus, purpura, pellagra, keloide, syphilitic eruptions, and diseases of the sebaceous follicles; these are separately described.

### EXANTHEMATA.

This name is given to acute inflammations of the skin, characterized by redness, which disappears during pressure, generally accompanied by constitutional symptoms; the classes are erythema, erysipelas, roseola, rubeola, scarlatina, and urticaria. The definition of Willan agrees with this; but he adds that the redness is sometimes attended with partial extravasation, and he admits purpura; this is placed by M. Biett among the anomalous orders, and the reasons for the alteration will be given where the disease is described.

*Erysipelas.* Willan places this among bullæ; but, as the vesications are considered by M. Biett to be complications, the agreement of the other symptoms with exanthemata induces him to place it under this order. This agrees with the opinion of Mr. Lawrence, who thinks that erysipelas should not be separated from erythema, but that it should follow it, in a natural nosology (*Medico-Chirurgical Transactions, vol.* XIV. *p.* 36). Of erysipelas, which they define as a non-contagious exanthema, characterized by a deep red colour of a considerable portion of the skin, with heat and tumefaction of this membrane, as well as of the subcutaneous cellular tissue, two species are admitted—1, True; and, 2, Phlegmonous erysipelas, as these agree with the E. simplex and phlegmonosum of Lawrence, which have been minutely described by that author, and reviewed in a former Number of this Journal, we shall not again detail them. M. Biett does not even go so far as Mr. Lawrence in recommending tonics or stimulants in any cases; the treatment to be pursued is to be strictly antiphlogistic. In true erysipelas of small extent, unaccompanied by general symptoms, low diet, with diluent drinks and lotions of Goulard water, are sufficient. Where the extent of the inflammation is greater, and the general symptoms are severe, with inflammatory fever, particularly where the individual is young and plethoric, blood is to be taken from the arm as often as the symptoms require it. When the pulse loses its strength, local bleedings are to be substituted. It is also serviceable to employ simultaneously local and general bleedings, taking care to take blood only from the neighbourhood of the inflamed surface, never from that itself. Where the patients are debilitated and aged, great caution must be employed in using any of these remedies. Laxatives and mild purgatives are to be employed; more drastic purgatives and emetics are not to be given if there is much fever, dryness of the skin, heat in the epigastrium, with great thirst. Blisters should never be employed, except to fix the erratic erysipelas, and to recal the eruption if it suddenly disappears. This is contrary to the opinion of Baron Dupuytren, who applies blisters to the inflamed surfaces in all cases of erysipelas; considering the disease to be an effort of Nature to throw out a morbid matter, he endeavours to assist Nature, by producing an artificial crisis by vesications; he has found this treatment particularly successful in erratic erysipelas, which never forms bullæ.

The other exanthematous diseases are correctly described; but, as the divisions are similar to Willan's, and the treatment the same as is followed in this country, we shall pass to the next order.

### VESICULÆ.

M. Biett admits five classes—varicellà and miliaria, which are essentially acute diseases; eczema, herpes, and scabies, which are most frequently chronic. This division is important, as it regards the treatment, the two former requiring, in general, only a simple antiphlogistic regimen, the three latter are more obstinate, often demanding very energetic remedies. Willan adds vaccinia and rupia. M. Biett considers that the former may be more conveniently classed with pustules, and that the latter is a " bulla."

*Eczema.* This is an eruption of very small vesicles, agglomerated in large numbers, and usually occupying extensive, irregular, uncircumscribed surfaces. M. Biett has been in the habit of dividing, in his lectures; eczema into acute and chronic.

1. *Acute Eczema.* This comprehends (1) Eczema simplex; (2) E. rubrum; (3) E. impetiginodes.

(1). *Eczema Simplex.* This consists of extremely small vesicles, situated closely together, and developed without any areolar inflammation, on a surface, the colour of which does not differ from that of the surrounding skin; the vesicles are transparent and brilliant, they are formed without any precursory symptom, the patient finds a slight itching, and is surprised to see a numerous crop of vesicles; the small drop of serum they contain becomes disturbed and milky, it is absorbed, the vesicle dries up and desquamates. This eruption does not produce those inflamed surfaces, that exhalation of serosity and constant renewal of scales which are observed in the other varieties. Its progress is slow, and the successive eruption of new vesicles extends its duration generally to two or three weeks, sometimes more. It may be general, but more frequently it is circumscribed, and confined to parts of the arm and fore-arm, or between the fingers, where it may be mistaken for itch. Young people and women are most subject to this slight eruption; it can frequently be traced to arise from the direct application of irritants, as heat, medicated ointments, &c.

Acute eczema is, in the greater number of cases, a more severe complaint than E. simplex, and this more acute form has two well-marked degrees of intensity, namely, E. rubrum and E. impetiginodes.

(2). *E. Rubrum.* This eruption is preceded and accompanied by heat and tension; the skin is inflamed and of a bright red colour. If it is closely examined, it appears to be studded with small silvery eminences; these are the commencement of vesicles, which, in a short time, acquire their full size, and appear about the size and form of the head of a small pin, with a well-marked inflammatory areola. From the 6th to the 8th day, sometimes before, the redness diminishes, the fluid is absorbed, the vesicles dry up, and the disease terminates by a slight exfoliation, produced by the " debris" of the vesicles. On examining the eruption at this period, it presents some characteristic marks, the surface is of a reddish colour (which remains for some days after the cure), covered with small round points; each of these is exactly surrounded by a whitish border, indicating the line of demarcation between the portion of epidermis, which was raised, and the areola which surrounded its base. At other times, the termination of E. rubrum is not so simple; the inflammation, instead of diminishing, persists, or even augments, the vesicles become confluent, break, and allow the fluid they contain to escape—this, flowing over an already inflamed surface, irritates it more, and causes superficial excoriations, which pour out a serous liquid; this thickens, and forms thin and soft lamellæ, often of considerable size, which are frequently renewed. The serous exhalation gradually ceases, and the healing process proceeds from the circumference to the centre, the disease terminating in two or three weeks. Frequently the eruption, instead of gradually improving, goes into a chronic state.

(3). *Eczema Impetiginodes.* In the earliest stage, the vesicles are similar to those of E. rubrum, but the inflammation is more acute, the skin is tumefied, the liquid contained in the vesicles loses its transparence, and becomes sero-purulent. These pustular vesicles, agglomerated and confluent, burst, the liquid concretes, and forms, not lamellæ, as in E. rubrum, but yellow, soft scales, composed of numerous thin layers, sometimes very large. These fall, and expose a crimson surface, from which a reddish fluid is poured out, and new scales are formed, which follow a similar course to the former ones; at length the inflammation diminishes, the pustular vesicles cease to be produced, the scales become thinner, the surfaces beneath them are less red, and the skin is gradually restored to its healthy state. This eruption may continue two or three weeks; it may be confined to a single spot, or cover the whole cutaneous surface, accompanied by severe general symptoms; when this is the case, the vesicles can often be seen in their various stages. When E. impetiginodes is confined to a single spot, a careful examination will detect the vesicles of E. rubrum around the vesiculo-pustular eruption, and in the centre of it. E. impetiginodes may pass into the chronic state, which does not differ from the chronic form of eczema rubrum. It must be clearly understood that E. impetiginodes is not E. rubrum complicated with pustules of impetigo, but an eruption of vesicles, at first transparent, which do not change into true pustules, but into pustular vesicles—otherwise the disease would be a true impetigo. Sometimes, indeed, the inflammation is so acute, that pustules of impetigo and of ecthyma are complicated with this eruption; but they are readily distinguished from it by their containing pus from the commencement, and from their larger size.

2. *Chronic Eczema.* This is the latter stage of the more acute forms of eczema. The skin, constantly irritated by the presence of an ichorous fluid, and by the successive eruptions of vesicles, instead of returning to its natural state, inflames very deeply, becomes excoriated and fissured, especially near the articulations; the constant exhalation of serosity is so abundant, that the linen covering the part is speedily soiled, and great care must be taken in removing it, lest the tender skin is torn, causing considerable hæmorrhage; the surface is tumefied and softened, so that the impression of the covering is often left upon it. In other cases, after a certain time, the fluid is exhaled in less abundance—it thickens and forms thin, soft, yellow lamellæ, slightly adherent, often of considerable size, leaving beneath them, after their fall, a surface inflamed and but slightly tumid. These lamellæ are formed more slowly, they are more dry, and the disease seems on the point of being cured, when, without any known cause, the inflammation commences with fresh violence; the surfaces begin again to grow red, they are covered again with vesicles, which speedily break, and the disease follows the same progress which has been pointed out: this disease may last many years, with similar exacerbations, Finally, in some cases, all serous exhalation ceases, the scales become more dry, less yellow, and more adherent, the skin is red, thickened, and is marked with deep fissures. In this state, the eczema resembles some forms of psoriasis, a true squamous eruption, and the more so as the scales appear to be lamellæ of an altered epidermis, not the concretion of an exhaled fluid. Thus some cases of eczema become real scaly diseases, and the vesicular character returns, in proportion to their progress towards a complete cure. In some cases, especially on the limbs, there remains one or two small spots of skin, thin, smooth and shining; they are covered with whitish, and very small scales, similar to epidermis; no vesicle can be seen on these patches, and the diagnosis becomes very difficult, unless a new eruption over the surface, or some small vesicles around the circumference point out the nature of the eruption. Chronic eczema, at first occupying but a small surface, may extend very considerably; in some rare cases this eruption, which was at first no larger than a five shilling piece, has been known gradually to cover a whole limb. Every form of chronic eczema is attended with most severe itching, frequently more difficult to bear than acute pain. Whatever may be the courage of the patient he cannot resist the unconquerable desire he has of scratching the part, which only increases the symptom. The itching becomes intolerable and the cause of severe anguish, when the eruption occupies the upper and inner parts of the thighs, (in women often accompanied by a chronic discharge from the vagina) and extending around the vulva and anus. After an indefinite space of time, the itching becomes less, the serous exhalation diminishes gradually, and at length ceases; the scales are more dry, the skin less inflamed, the circumference is the first cured, the lamellæ become thinner and smaller until they cease to be formed, the skin remains for some little time after the eruption has disappeared, rather redder and smoother than natural, but is eventually restored to its natural state. The duration of this eruption is indefinite; it may remain many months or even years.

Eczema may arise on any part of the surface of the body, but its more frequent situation is on that part of the skin which is furnished with hairs and numerous follicles, as the pubis, groins, scrotum, &c. In some cases it is confined to a single point, as the breast, the hairy scalp, the ears, the scrotum, constituting local varieties of considerable practical importance; but most frequently it appears on many parts at the same time, so as sometimes to occupy the whole surface of the body simultaneously. Eczema is not contagious; but, in a few cases it has been communicated by the prolonged contact of two mucous surfaces during coition. It attacks adults, and women more frequently than men. The causes are in many cases not discoverable; in others they are easily traced; thus it is often the result of the heat of a large fire or of the rays of the sun (E. solare—Willan); it follows the application of a blister, or of frictions, particularly with irritating ointments, as mercurial ointment. This has been called eczema mercuriale, but it differs from neither of the other forms. It is often produced by the excessive use of ardent spirits; and in those individuals who work in the sugar refiners' manufactories ecxema frequently follows a burn. Whatever may have been the causes which produced eczema, its chronic state and its duration generally for a long period must be attributed to a peculiar constitutional disposition. The handling of metals, the contact of powdery substances, &c. are the causes frequently of eczema of the hands (grocers' itch).

*Diagnosis.* This is of very considerable importance. Eczema simplex has often been mistaken for the itch, with which it agrees in being developed without inflammation, in affecting certain parts, as the wrist and lateral parts of the fingers, and in causing severe itching; but the vesicles of eczema are flattened, those of itch are acuminated; those of eczema are always agglomerated, in itch they are in general isolated or altogether distinct, or but one or two vesicles are observed in a surface of some extent, as between two fingers, which is never the case in eczema. The itching of eczema is a kind of smarting very different from the exacerbations of itching in the itch, in the former case it is truly painful, whilst in the latter it is a sensation "plutôt agréable que pénible"! Finally, the itch is contagious, eczema is not generally so. Eczema rubrum may be confounded with miliaria, but in this latter eruption the vesicles are never confluent as in the former, or confined to a small space, they are on the contrary in an innumerable quantity; they are more voluminous in miliaria; the severe general symptoms which accompany symptomatic miliaria, sufficiently distinguish this affection from eczema. That variety of miliaria which appears in individuals who have taken severe exercise during the excessive heat of Summer resembles eczema considerably, but the vesicles are more universal, the sweats are most abundant, and the termination of the eruption more speedy than in eczema. Eczema impetiginodes differs from impetigo by well marked characters. The vesicular eruption covers large surfaces, impetigo is most frequently confined to a small space. The pustules of impetigo never contain even at their commencement a transparent serum, their base is larger and the fluid they contain is thicker. The pustular vesicles of eczema impetiginodes are always vesicular at their commencement, they never contain true pus but a yellowish serosity, a sero-purulent liquid. In impetigo the pustules produce true scabs, which are thick, more or less yellow, ragged, unequal, like shagreen; whilst the pustular vesicles of eczema only form thin scales, of greater extent than thickness, and around them vesicles of eczema rubrum are always seen, which never is the case in impetigo. When the vesicles of itch are mixed with pustules (which are in the greater number of cases a complication) this eruption may be confounded with eczema impetiginodes, unless the vesicles themselves which always exist in large numbers are not carefully examined according to the diagnostic marks stated above. The diagnosis of chronic eczema is often much more difficult. In lichen agrius there is an exhalation of serosity which forms scales, but these scales are smaller, thicker, and more yellow than those of eczema, approaching more nearly the nature of scabs; if they fall they expose, not a red, smooth, shining, and slightly excoriated surface as in eczema, but a surface irregular from a number of small prominent points (papula) generally appreciable to the eye, and constantly to the finger gently drawn over the seat of the eruption. At other times, as in chronic eczema, the lichen produces thin dry scales without any appreciable serosity and local inflammation, but then the skin is much thicker and ragged than in eczema. In lichen some few papulæ are constantly found near the eruption, easily recognised by their hardness and chronic progress, whilst vesicles are seen near the patches of eczema. It requires great attention to distinguish these two eruptions on the hands. Some varieties of chronic eczema may be mistaken for psoriasis; but they differ from it in the constant presence of vesicles in the neighbourhood of the eruption and in the scales which are less dry and less friable. In that form of chronic eczema in which at a late stage the skin is red and covered with small whitish scales, the diagnosis is much more difficult, particularly if the physician has not watched the progress of the disease; but the appearance of the skin is different in the two affections; in eczema it is not elevated or in a state of hypertrophy as in psoriasis, neither is it marked with fissures in every direction as in psoriasis inveterata, but they are in relation to the muscular motions of the part.

*Prognosis.* Eczema, particularly the acute form, is generally a mild eruption, but when it becomes chronic and occupies a considerable surface, it is a very troublesome disorder. The prognosis is more unfavourable when the eruption has existed many years, and new crops of vesicles form when a cure is expected. Without endangering the life of the patients it embitters their existence.

*Treatment.* In the slight forms of eczema simplex a mild diet, lemonade for a beverage, and the local application of warm water is sufficient; when the eruption is prolonged, and of greater extent, attended with severe itching, laxative drinks must be given and alkaline or sulphur baths employed. The alkaline baths are made by dissolving in the bath from four to eight ounces of subcarbonate of potash or of soda, according to the age of the patient and the stage of the eruption: and in a sulphur bath, four ounces of sulphuret of potash are to be dissolved. If eczema rubrum and impetiginodes occupy but a small surface, the patient must be put on low diet with diluent drinks; if they are of greater extent, and the constitution sympathises, especially if the patient is young and vigorous, either a general or local bleeding, by means of leeches, in the vicinity of the eruption, must be resorted to, with simple and emollient baths; or local mucilaginous baths and potato cataplasms, if the vesicles have broken, and have left the surface red, excoriated, and painful; applications containing sulphur or mercury are to be avoided as likely to increase the irritation. In all cases it is obvious that the irritating causes must first be removed. Chronic eczema, when not very far advanced, often yields to the following treatment: acidulated drinks and baths are the remedies most generally employed. Half a drachm to a drachm of nitric or sulphuric acid is to be mixed with a pint of barley water, and drank at intervals in small quantities, a small cup of cold water being taken after each dose of the acid at first until the stomach becomes accustomed to it. This is particularly beneficial where there is great discharge of serum from the surface of the eruption with intense itching. Nitric acid is more energetic than the sulphuric. The heat of the baths should be from 25° to 27° (Réaumer), the patient should remain in them about an hour daily; they are rendered emollient by the addition of mucilage and of gelatine—a pound and a half of gelatine is necessary for a single bath. Laxative drinks are frequently useful, as a pint of whey, in which two drachms of acidulated tartrate of potash has been dissolved, or half an ounce of sulphate of soda or potash mixed with a pint of veal broth, &c. Alkaline remedies are very serviceable when, notwithstanding the use of emollients, the itching continues unabated; the alkaline bath should be taken before going to bed. From half a drachm to a drachm of subcarbonate of potash may be daily administered internally. When the eruption is of longer standing and occupies a more extended surface, more active remedies must be employed, such as purgatives, sulphur baths, and vapour baths. The purgatives to be employed are calomel, Plummer's pill, aloes, jalap, gamboge, and Seidlitz water. Sulphureous waters can be taken internally as well as applied externally; they are only suitable when the disease has existed for a very considerable time, and when the eruption affecting the lower limbs is of a violet colour. The waters of Barèges, of Enghien, and of Cauteretz are the most usually employed; they can be produced artificially, by adding to a simple bath two or three ounces of sulphuret of potash, varying the quantity according to the excitement which is desired to be produced; in all cases it is proper to use alternately simple warm baths with sulphur baths. When these waters are administered internally they should be diluted at first with two thirds barley water or milk, and this should be gradually diminished until the patient can take the medicine in its undiluted form. Vapour baths are sometimes very useful, but care should be taken that the patient does not expose himself to very hot vapour. When the disease is local, a stream of vapour directed on the part (les douches de vapeur) is often very serviceable. If the extent of the eruption is much reduced, its cure is hastened by gently rubbing the part with an ointment composed of a scruple or half a drachm of the ammoniated protochloride of mercury and an ounce of lard. In the course of the treatment intense itching is relieved by the application of lotions containing superacetate of lead, emulsion of bitter almonds, or decoctions of dulcamara, hyosciamus, &c. In more obstinate cases energetic remedies must be employed, provided there is no chronic affection of the stomach or intestines, such as tincture of cantharides, commencing with a dose of three drops every morning, then five drops, and augmenting it five drops every six or eight days until each dose contains twenty-five or thirty drops, and arsenical preparations, small doses of which are to be continued for a month or six weeks. In the scaly form of eczema, when local, particularly when occupying the hands, the following applications are useful. Ointments, composed of protonitrate of mercury, ℈j. to an ounce of lard; or, of protoioduret of mercury, ℈j. or so, or twelve grains of the deutoioduret of mercury to the same quantity of lard; a little camphor may be added to appease the itchings; the advantages of the internal use of mercury are at least doubtful, sometimes it is injurious.

Among the local varieties it is of importance to mention, eczema of the hairy scalp. In some cases it occupies this part alone, but most usually part of the face also. The exhalation of a fluid is so profuse that the hairs are at first soaked in it; the serum dries and forms whitish scales which cover and connect the hairs separately in small bundles of five or six hairs each; from this circumstance and the colour of the scales Alibert has termed it teigue amiantacée. When the exhalation is less abundant the scales are smaller, dry, and furfuraceous, quickly renewed, falling off in great abundance if the slightest friction is used; this is the teigue furfuracée of Alibert, and may be confounded with pityriasis capitis. The bulb of the hair is not diseased in either of these affections, and they require a simple and mild treatment, such as slight laxatives, emollient washes at the commencement, and alkaline lotions, when the eruption is farther advanced. By those who do not pay sufficient attention to primary forms of eruptions, this has been confounded with porrigo; but the diagnosis is simple and of the highest importance. As eczema is one of the most frequent, and in its chronic form, one of the most tedious, cutaneous eruptions, we have given a lengthened and detailed description of it, particularly as M. Biett has paid especial attention both to its diagnosis and treatment, which, together with the division which he has adopted, into acute and chronic, are of much practical value. The Synopsis of Bateman, which cannot be too highly praised for the correct and classical descriptions which it contains, and the medical learning and research which it exhibits, is often deficient in diagnosis; this point has been carefully supplied by our authors, and we shall make much use of it.

*Herpes.* This is divided into, 1, Herpes phlyctenodes, having two varieties—H. præputialis and labialis—2, H. zoster—3, H. circinatus—4, H. iris. Neither the description of these forms, nor their mode of treatment, differs from that given by Bateman.

*Scabies.* M. Biett considers this to be a decidedly vesicular disease, and classes it under this order. Bateman says, " that from its affinity with pustules, vesicles, and papulæ, it almost bids defiance to an artificial classification;" and, without any sufficient reason, he follows Willan, and classes it among pustulæ, although two of its varieties, S. papuliformis and S. lymphatica, are truly vesicular, and much more frequent than S. purulenta, a comparatively rare form of the eruption. The term S. papuliformis, which Bateman applies to his first variety, is indefinite, as the eruption consists of " minute itching vesicles" *(Bateman's Synopsis, p.* 163, *6th ed.)*, resembling papules only when carelessly examined, it is objectionable, as liable to confuse the student, who should constantly bear in mind the distinct vesicular nature of scabies, in contra-distinction to the papulæ of lichen and prurigo, which are often mistaken for it.

The itch is a contagious eruption. In infants, it is developed four or five days after exposure to contagious influence; if they are very feeble, a few days longer—if very strong and robust, not more than two days intervene; in adults, from eight to ten days in the Spring and Summer—from fifteen to twenty in the Winter; it is longer in old men, whose skin is more dry and hard.

*Symptoms.* A slight itching is first felt in the parts which have been exposed to the contagious contact, increasing by the warmth of the bed, or by heating foods; accumulated transparent vesicles form, of a light rosy hue, in young subjects, containing a serous and viscid liquid. If the patient is weak, their progress is slow, and vice versâ. The eruption appears in the flexures of the joints, as at the wrist, between the fingers, in the bend of the arm, arm-pit, hams, &c. and, later, on the belly. It may occupy the whole cutaneous surface, with the exception of the face; but more often it is less extensive. In some cases, the number of vesicles is very few, and they are scattered; if they are numerous, the itching is so insupportable that they are destroyed by scratching, the liquid escapes, and the vesicles are re-placed by numerous red and often inflamed points, and thin, yellowish scales. If the patient is young, vigorous, and of a sanguine temperament, the action of the nails may so augment the inflammation, that the eruption becomes complicated with pustules of impetigo and ecthyma. In some few cases, the vesicles have become pustular.

Although M. Biett has employed, at various times, the strongest microscopes in investigating this eruption, he has never been able to discover the insect, which various writers have described with minuteness, and of which M. Gales, in 1812, stated that he had collected upwards of 300, and was enabled to ascertain their mode of generation, laying eggs, &c.! M. Biett believes that they do not exist; it would be more charitable to these writers to consider with Bateman, who was unable to discover these insects, that the production of acari, in scabies, is a rare and *casual* circumstance.

*Diagnosis.* It is hardly necessary to mention the importance of accurately distinguishing this disease, both for the comfort of whole families and the reputation of the medical practitioner. It is often confounded with prurigo; but, independently of the distinct primitive characters, the former vesicular, the latter papular, scabies is found in the flexures of the joints, whilst prurigo occupies the opposite surface, "dans le sens d'extension." In prurigo, the summit of the papulæ, which are almost always destroyed by scratching, has a small, dry clot of blood, black, or nearly black; the broken vesicles of itch are covered by a small, thin, yellowish scale; in prurigo, the itching is more severe and burning, and the eruption is not contagious. Lichen simplex may sometimes be mistaken for scabies; but, on examination, it will be found to consist of papulæ, generally very close together, which is never observed in the itch; these papulæ are of the colour of the skin, whilst the vesicles of itch are slightly rosy; when lichen affects the hands, it is found on their backs, not between the fingers; the itching is slight, the eruption is not contagious. The vesicles of eczema are flattened, of scabies acuminated; in eczema they are agglomerated, in scabies generally distinct; the itching of eczema is a general smarting, very different from the exacerbations in itch. Eczema is non-contagious. Scabies may be complicated with eczema; this is often the effect of the irritating applications of quacks; also with the pustules of impetigo and ecthyma, which appear on those parts where the vesicles exist in great numbers; and these are the cases which have been called pustular itch (scabies purulenta). If the inflammation increases, it may affect the cellular membrane, and these pustules may be complicated with boils. In some few cases, it exists simultaneously with lichen.

*Prognosis.* Scabies never terminates spontaneously; abandoned to itself, it may remain many years, or a whole life. Under a rational treatment, its duration varies from ten days to one or many months, according to its complications.

*Treatment.* M. Biett has found the following remedies to succeed most constantly and promptly, without causing, from irritation, other eruptions. Half an ounce of an ointment, composed of two parts of sublimated sulphur, one part of subcarbonate of potash, and eight parts of lard, is to be rubbed over the parts affected with the eruption night and morning; a warm bath is to be used every day, or at least every other day. The average length of this treatment is twelve days. For young children, sulphur baths and ablution with soap and water are alone necessary. Sulphur baths and fumigations are useful auxiliaries, but the cure is tedious (from 25 to 33 days) if they are alone employed. The " lotion of Dupuytren" is conveniently employed, in those cases in which the patients do not like frictions with ointment; but it does not suit irritable skins. It consists of four ounces of sulphuret of potash, half an ounce of sulphuric acid, and a pint and a half of water; sixteen days is the average length of time required for a cure. In recent and mild cases, the powder of pynovel, which is powdered sulphuret of lime, is serviceable; half a drachm of the powder, moistened with a little oil, is to be rubbed over the eruption night and morning. The cure is effected in about 15 days. If the itch is complicated with eczema, the sulphur applications must not be employed, but diluent and slightly acidulated drinks must be substituted. If there are pustules of impetigo or ecthyma, the use of any irritating application must be discontinued; the simple warm bath and gently laxative drinks are useful, and it is advantageous to soak the hands and fore-arms, which are often the seat of the eruption, in local emollient baths. In all cases, the linen should be frequently changed.

*Bullæ.* The two varieties of this order are pemphigus and rupia. Pemphigus consists of an eruption of bullæ, of variable size, on different parts of the body, containing a liquid, at first serous, subsequently reddish; they are usually distinct, but numerous, succeeded by thin crusts and superficial excoriations. There are two forms, the acute and the chronic.

(1). *Acute Pemphigus.* The eruption is preceded, during 24 or 48 hours, by symptoms of fever, or, in milder cases, by a general feeling of malaise. Circular patches of a red colour are seen, distinct, small, and numerous, generally scattered over a large extent of the surface of the body; on these, bullæ are speedily formed; when they cover the whole inflamed surface, they have the appearance of small, isolated, transparent tumours, from the size of a pea to that of a nut; but when they only occupy the middle of the inflamed spot, there is a red areola surrounding each. In some cases, bullæ do not form on all the erythematous patches, but they are speedily produced on rubbing the part. After some days they open, and are replaced by thin, brown crusts, or little dry whitish lamellæ. Sometimes the attendant general symptoms are very slight. The duration of the disease is from one to three weeks. Dr. Willan denied the existence of an eruption of bullæ with fever, to which the older writers had applied the term pemphysis; and defined pompholyx as "an eruption of bullæ, without any inflammation around them, and without fever." The pompholyx diutinus of Willan, answers to the chronic pemphigus of Biett. It is more common than the acute form, and is confined to adults and elderly people. Feverish symptoms are not observed unless the eruption is very extensive, and prolonged by successive crops of bullæ. It is preceded by lassitude and pains in the limbs; numerous red points first appear, attended with a tingling sensation; the epidermis becomes raised by a serous fluid, forming, in a few hours, irregular bullæ, as large as a nut or a walnut; these, in a few days, increase to the size of an egg or larger; they break, and discharge a lemon-coloured serosity, and the epidermis, relaxed and wrinkled, is partially detached, exposing an excoriated painful, and red surface; towards the third or fourth day, the liquid, in the bullæ which have not burst, becomes reddish, the vesications subside, and small, brownish, thin, and flattened scabs, are formed. New bullæ are formed near the older ones, and they go through the same stages. In some rare cases, the whole surface of the body is covered with the bullæ, which become confluent. The eruption continues from one, two, or three weeks to many months or years. It most frequently attacks old people, who have been debilitated by the effects of bad and scanty diet, damp situations, &c.

*Diagnosis.* From rupia simplex, by the bullæ in this affection being few in number, and followed by true ulcerations and thick prominent scabs. In ecthyma, the epidermis is sometimes raised so considerably by pus, as to be mistaken for a bulla, but the contents are purulent, not serous. When the bullæ of pemphigus are of small size, and near together, it resembles herpes phlyctenodes; but distinct bullæ in other parts, and their size being more considerable than that of vesicles where close together, mark the difference. The bullæ on an erysipelatous surface are accidental productions. The scabs of pemphigus may be confounded with those of impetigo; but, in the former eruption, they often cover the body, or nearly so; in the latter, they are generally confined to a smaller surface. The scabs of impetigo are rough, thick, and irregular; those of pemphigus thin, often convex in the centre, and the shape of the large bullæ.

*Prognosis.* The acute form is a mild disease, and terminates favourably; the chronic always indicates a bad state of the constitution, and is dangerous, if the eruption is very extensive, and occurring in individuals weakened by old age, misery, or debauchery.

*Treatment.* In acute pemphigus, strict diet, diluent drinks and rest are sufficient, unless inflammatory symptoms exist; in such cases, tepid baths, bleeding from the arm, or leeches to the anus are required. Chronic pemphigus requires a cautious antiphlogistic treatment, diluent and acidulated drinks, tepid baths, and, in a later stage, alkaline baths; if there is much pain, soothing applications, and opiates administered internally; bleeding will be required, if there are symptoms of inflammation of the lungs, or any internal organ. If, notwithstanding the use of such remedies, new crops of eruption appear, the strength of the patient must be restored by good nourishment, wine, acids, and quinine, &c. with preparations of iron and other tonics; this change of treatment is of advantage, even in young subjects.

*Rupia.* Biett has differed from Bateman, who placed this eruption under vesiculæ. It is defined as an eruption of bullæ more or less voluminous, isolated, flattened, filled with a fluid sometimes serous, sometimes purulent or blackish, succeeded by thick scabs and ulcerations. It is divided, according to Bateman's classification, into R. simplex, prominens, and escharotica. The ulcerations frequently heal slowly, and it is recommended to quicken the process of cicatrization, by the gentle application of nitrate of silver; in some cases, it becomes indispensably necessary to modify the diseased surface by means of active caustics, such as the repeated and lengthened application of nitrate of silver, or of lotions of nitric or muriatic acid; in some cases the concentrated acid is necessary. M. Biett has found an ointment composed of one scruple of the proto-ioduret of mercury, or of twelve to fifteen grains of the deuto-ioduret to an ounce of lard, very efficacious.

### Pustulæ.

*Impetigo.* This is characterized by an eruption of psydacious pustules generally situated closely together, and forming thick, rugose, and yellowish scabs. From the form these pustules assume, it is divided into impetigo figurata, having a determined form, either round or oval, and I. sparsa, in which the pustules are irregularly disseminated. The impetigo scabida of Willan is that state of impetigo figurata in which the scabs have acquired a considerable thickness. The description of these forms given by Bateman is not excelled by our authors.

*Diagnosis.* The psydracious pustules forming thick, ragged, yellowish scabs, distinguishes the eruption from the vesicular or pustulo-vesicular eczema which forms thin scaly lamellæ. When impetigo is situated on the chin it is likely to be confounded with sycosis, but in impetigo the pustules are small, yellow, and close together, the scabs of a thick transparent, yellowish green; there are neither callous elevations, or tubercles; the pustules of sycosis are larger, less yellow, isolated, more elevated than those of impetigo, the discharge is much less abundant, and the scabs are drier and of a deeper colour. The distinct pustules of porrigo, "let into" (endrâssé) the epidermis forming speedily a yellow and dry scab, of a cup shape, as well as the contagious nature of the eruption and the loss of hair which accompanies it, and which never follows impetigo, distinguish porrigo favosa and scutulata from any impetiginous eruption.

*Treatment.* When the eruption is slight, emollient lotions with decoction of poppies, tepid milk, almond emulsion, &c. and cooling drinks, are sufficient; if it is more extensive with general symptoms, bleeding either local or general, and tepid baths; towards the end of the disease, and particularly when it is obstinate, vapour and douche baths, together with active purgatives of calomel, jalap, Epsom salts, &c. or acidulated drinks, with alkaline alternated with acid baths, or a lotion composed of medicinal hydrocyanic acid, ʒij. vel. ʒiij. alcohol, ℥ss. distilled water, ℥viij.; previously to the employment of these applications it is necessary to cleanse the surface from the scabs. When the eruption becomes chronic, sulphur taken internally, and applied externally by means of baths, is very beneficial. In those cases which have resisted all other means of treatment, a strong solution of nitrate of silver or an acid has been applied to the diseased surface, and immediately washed off, so as to prevent the too great action of the caustic. In such cases the internal employment of small doses of arsenic is of signal service.

*Acne.* A chronic pustular affection, consisting of an eruption of small, isolated pustules, of a deep red colour, having a base more or less hard, and frequently forming after the disappearance of the pustules, small, hard, red, circumscribed, indolent tumours. It will be seen by this definition that the tubercles which were regarded by Willan as the elementary lesion are here considered to be the termination of pustules. Acne punctata, which is admitted by Willan, certainly does not agree with his definition of acne; it is omitted by M. Biett, who considers it only as a frequent complication. The division into A. simplex, indurata, and rosacea, is adopted. The descriptions of this well-known eruption do not differ materially from those by Bateman; but as there are some peculiarities in the mode of treatment, we shall mention them; few cutaneous diseases being more troublesome from their obstinacy in resisting medical aid, and more annoying from their affecting those parts which are most exposed at an age when the "cura cultus sui" is considered of much importance. When there is a considerable crop of pustules of acne simplex, or indurata, a general bleeding, low diet, with abstinence from spirits, wine, &c. is necessary; to produce an absorption of the tubercles, lotions of rose-water and alcohol should be employed, their strength depending on the state of the pustules, and the quantity of irritation which is required to be produced, or a lotion containing five or six grains of oxymuriate of mercury, an ounce of alcohol, and half a pint of distilled water; frictions also are very useful, with an ointment composed of the ammoniated protochloride of mercury, ℈j. to ʒj. lard, ℥j. But of all the preparations which M. Biett has employed, he has found none more useful in producing the absorption of these tubercles than the ioduret of sulphur ointment, (ioduret of sulphur, gr. xij. vel. gr. xxiv. lard, ℥j. Baths, and particularly douche baths, of the vapour of hot water, directed on the face for twelve or twenty minutes daily, assist the action of the other remedies. Purgatives are hurtful. The sulphur waters of Barèges, Enghien, &c. are useful, both taken internally and employed externally. In acne rosacea, these local applications are generally useless, often prejudicial; great attention to the state of the general health is alone required; excesses at the table, wine and spirits, are to be avoided; the habits of the patient must be sober and regular; a mild diet habitually composed of white meats, fresh vegetables, cooling succulent fruits, must be persisted in, and constant care taken to avoid fatiguing exercises of all kinds, confinement to the house, or in warm rooms near a fire, mental anxiety, &c.

*Sycosis or "Mentagre."* This, like acne, is considered by Biett to be pustular in the first instance, and to be tubercular consecutively. Its seat is principally the chin, upper lip, and occasionally the lateral parts of the jaw over the masseters. It commences by distinct, accumulated, psydracious pustules; in six or seven days these burst, and brownish scabs are formed, or if the subject is young and vigorous, one large scab. The centre of each pustule will be found to be occupied by a hair. In many cases, particularly in weak and feeble individuals, as the pustules decline, their base becomes hard, red, and tumefied, complete resolution not having taken place; from this tubercular appearance sycosis has been mistaken for an originally tubercular disease. The summits of these elevations may subsequently become ulcerated. Its duration is very variable; in many individuals it is very obstinate and subject to return.

*Diagnosis.* The pustules of impetigo figurata are flattened, and hardly elevated above the level of the skin; they are in groups, and their progress is acute: in sycosis the pustules are acuminated, elevated above the level of the skin, generally isolated and distinct. The pustules of impetigo open about the third or fourth day, and the fluid that escapes forms bright yellow, thick and large scabs; the pustules of sycosis do not burst until the 5th or 7th day, and the scabs are of a deep brown colour, much thinner, and drier, than those of impetigo. The pustules of ecthyma are larger, and their base more inflamed, than those of sycosis; the size of the scabs is also greater, they are thicker, and more adherent, and not followed by tubercular indurations. Syphilitic pustules are distinguished from sycosis by the absence of heat and pain; their base is copper-coloured and violet, they are rarely found on the inferior part of the face, but rather on the alæ nasi, forehead, and commissure of the lips. Syphilitic tubercles are of a shining, and of a dull copper colour, appearing to affect the superficial layers of the skin, whilst the conoidal tubercles of sycosis are deeply implanted in it.

*Treatment.* In the first place the cause is to be removed. The beard is to be cut with scissors, not with a razor; repeated applications of leeches behind the ears, and below the jaw; emollient fomentations and poultices of bread or potatoe, and purgatives with low diet in the first instance. When the disease has lasted some time, and the tubercles are voluminous, stimulant frictions with the ammoniacal protochloride of mercury, or of the deutoxide or sub-sulphate of mercury, in the proportion of a scruple to a drachm of either of these preparations to an ounce of lard, are to be employed; also vapour-baths, and streams of vapour, or sulphuretted waters, directed on the part, renders absorption more active.

*Porrigo.* Seven varieties of this affection have been described by Willan. M. Biett thinks that there are but two kinds, which he names P. favosa, and scutulata; he considers porrigo furfurans to be either pityriasis capitis, or more often chronic eczema of the scalp; and porrigo larvalis to be a form of eczema impetigenodes, or impetigo figurata, to which also he refers the porrigo favosa of Willan, and describes under the term favosa, what that author has called lupinosa. The importance of readily distinguishing these contagious cutaneous affections is such, that any division by which their diagnosis is rendered more simple is very advantageous. Dr. Bateman felt the impropriety of classing contagious and non-contagious eruptions under the same class, and in a note to his 6th edition, (p. 160,) questioned the propriety with which Dr. Willan classed the crusta lactea under the genus porrigo, and confessed that he left it there "in deference to his venerated preceptor."

*Porrigo Favosa. (P. Lupinosa, Willan; Teigne Faveuse, Alibert.)* This eruption most frequently affects the hairy scalp, but it is sometimes found on every other part of the body. The eruption consists of pustules denominated "favi," which are at first small, circular, set deeply in the skin, containing a liquid which immediately concretes, forming a yellow, straw-coloured, dry matter, having a central depression, which can by means of a lens be recognized at a very early period; this is well-marked at the end of five or six days; the pustules are usually isolated at the beginning, sometimes grouped; the skin surrounding each is very red, there is greater or less itching, and each pustule is often traversed by a hair. The concretions augment slowly in quantity, preserving their central form and cup-like depression; when they approach each other, they unite and form yellow incrustations of greater or less extent, covered with alveolar depressions, each of which corresponds to a former pustule. Alibert has judiciously compared the appearance of this stage of the eruption to that of the cup-like receptacles of lichens, which cover the trunks of some trees; this is peculiarly applicable to these incrustations, which sometimes surround the limbs. If these concretions are detached by emollient applications, the skin beneath is found to be slightly eroded, and new incrustations do not form unless a renewed eruption of favi takes place; the red and fetid sanies which often flows from the excoriated surface forms irregular scabs. When the disease is not interfered with, the crusts become very adherent, and remain for months or even years; they increase in thickness, are of a white colour, and eventually break off in fragments; the skin beneath is thickened and changed by chronic inflammation. Frequently successive crops of pustules form in any places. The hairs even from the commencement are pulled out with facility; in a later stage they fall and leave the skin smooth and shining: new hairs rarely are formed on this surface; if they are re-produced, they have a remarkably lanuginous appearance. Alibert has compared the smell of the eruption to the urine of a cat. The duration of the eruption is uncertain. Alibert has denied the contagious nature of this disease, but M. Biett has been convinced, from numerous cases, that this opinion is not correct. A case which occurred in one of his wards at the Hôpital St. Louis last Summer, and which was reported at the time in one of the medical periodicals, afforded him an additional proof of its contagious nature. A man had been a patient in the wards some months, the greater part of whose trunk and legs were covered with the well-marked eruption of porrigo favosa. In the next bed was a patient admitted subsequently with pustules of ecthyma luridum, and after some weeks two or three well-marked "favi" appeared on his fore-arm. On inquiry it was found that this man officiated as tailor to the ward, and had been employed in mending some of the garments of the infectious patient.

*Treatment.* This is almost wholly external: in a few cases only tonic bitters or laxatives are required. Great cleanliness must be observed, the hairs must be cut short or shaved, and the incrustations detached in order to apply local remedies to the diseased surface. In order to detach the incrustations, large emollient cataplasms must be employed, and the surface must be washed with soft soap and warm water. Preparations of alkalies and of sulphur, and acid lotions, are the best local applications. In order to detach the hairs after the surface is cleansed, the scalp is to be anointed daily from five to ten minutes with an ointment composed of one or two drachms of subcarbonate of potash or soda, mixed with an ounce of lard; lotions for the same purpose are made by dissolving two drachms of this salt in a pint of water. A lotion composed of a drachm or two of sulphuret of potash with a pint of water, or the following lotion, (Barlow's lotion,) containing sulphuret of potash, ʒij. white soap, ʒiiss. lime water, ℥vij. alcohol ʒj. frequently is of great use in modifying the diseased action of the skin. Light sulphur douche baths, repeated daily, fulfil the same indication still better than simple lotions; and they have this advantage, they prevent the ointments employed remaining too long on the surface. Great care is required in the application of the remedies. The basis of the remedies employed by the Messieurs Mahon is an alkali, and the fame they have gained is owing greatly to their applying invariably the medicaments with their own hands; the number of successful cases which they have treated has been over-rated, as they make no distinction between porrigo and other less troublesome affections of the scalp, denominating them all "teigne." Weak lotions, containing muriate and nitric acids, have been much recommended. M. Biett has found an ointment, composed of a scruple or half a drachm of ioduret of sulphur with an ounce of lard, and rubbed on the parts affected night and morning, the most efficacious of all topical applications; in a few weeks so modifying the diseased action of the skin, as to prevent the formation of new pustules, and to cause a growth of hair. When the eruption is on the body and limbs, baths, especially those of sulphur, are useful. When the disease is local, and consists of a few scattered pustules, after detaching the scabs, the surface should be cauterized with nitrate of silver. In all cases great perseverance is necessary both on the part of the physician and the patient.

*Porrigo Scutulata, Ringworm of the Scalp, Scald Head.* This differs from porrigo favosa in the position of the pustules, instead of being distinct and isolated they are always in groups, forming circles, in the circumferences of which the small yellow favi are in greater numbers than in the centre. They are preceded by red circular spots, and are accompanied by severe itching. The incrustations are at first thin, subsequently they become thicker, and so extensive that in old cases the whole scalp appears covered with a thick "calotte," the circumference of which bears evident marks of the first form of the eruption, such as small but defined parts of numerous circles; there are no hairs except at the union of the scalp with the skin of the face, and these are thin and lanuginous, forming a kind of corona; the eruption itself at this stage does not present the cup-like depressions of porrigo favosa, but yellowish-grey, dry incrustations, which are detached in small portions, having the appearance of coarsely powdered mortar. When the disease is more partial the pustules are sometimes found in all their different stages. The description of the pustules of the porrigo favosa, in their early state, applies to these. The hairy scalp is the special situation of porrigo scutulata; at the same time it may be found on the forehead and neck. It is very rarely seen on the body, and when it apears there, it is generally the result of direct contagion.

*Diagnosis.* The only species of porrigo with which this eruption can be confounded is the porrigo favosa; it differs from the others (so called by some authors) by the nature of the pustules (favi), the colour and form of the incrustations, the baldness which it produces, and its contagious character. The primary pustules of porrigo scutulata are like those of porrigo favosa, small, yellow, set deeply in the skin, and depressed in the centre; but in the former the pustules are agglomerated into circles, whilst those of the latter are at first distinct, and when united never form regular figures. When the incrustations of porrigo scutulata cover the head they are more likely to be confounded with a similarly extensive eruption of porrigo favosa, but the large incrustations of the latter are never bounded by defined margins, and if carefully examined some cup-like depressions will be found: whilst defined portions of circles are always seen around the circumference of the eruptions of the former, there are no cup-like depressions, and the dry and detached pieces of the crusts have the appearance of broken mortar. Impetigo figurata of the hairy scalp may be mistaken for porrigo scutulata, or this latter eruption on the limbs may be confounded with the former; as the pustules of impetigo figurata are agglomerated, forming thick scabs, regularly circumscribed, and sometimes circular; but the pustules of impetigo are superficial, slightly prominent, placed on a red and very inflamed surface, containing a liquid which slowly thickens, and forms a true scab at the end of some days; whilst those of porrigo scutulata are more deeply set in the epidermis, accompanied by a slight inflammation at their base, and containing a matter which is concrete almost at their first appearance; again, in a later stage, the scabs of impetigo are much thicker, and after they have fallen off are reproduced by a sero-purulent discharge from the inflamed surface, whilst the incrustations of porrigo scutulata are never reproduced unless by a new formation of favi. Impetigo figurata usually occupies but a small surface, the patches are isolated, it is not attended by loss of the hair, it is not contagious; porrigo scutulata is but rarely seen on the limbs, and then it generally is found at the same time on the hairy scalp.

The treatment to be pursued is precisely similar to that recommended for porrigo favosa. As it is highly contagious care must be taken that the cloths and sponges employed should not be used by uninfected individuals.

*Porrigo Larvalis of Willan, (Teigne Neuqueuse, Alibert.)* This non-contagious eruption, the crusta lactea of authors, has been well described by Bateman, who considered that it should be denominated impetigo larvalis. M. Biett considers it to be a form of impetigo, or eczema impetiginodes.

*Treatment.* In the greater number of cases tepid and emollient washes are alone required; in young infants it is sufficient to direct the nurse to eject the milk from the breast itself on the diseased surface. If the child is older, and there is much inflammation, a few leeches behind the ears; if it occupies the scalp, the hair is to be cut short, and the scabs detached by bread and milk or potatoe poultices. If it has become chronic, lotions containing a drachm of sulphuret of potash, and two drachms of sub-carbonate of potash or soda in a pint of water are useful in modifying the state of the skin. When it affects the trunk, sulphur baths, or sulphur douche baths are beneficial. Gentle laxatives may be given.

### PAPULÆ.

M. Biett recognises two forms of papulæ, lichen and prurigo; he conceives strophulus to be a lichen which affects infants; and as Dr. Willan has divided them merely from the one form affecting children, and the other adults, not from any distinction in their primary forms, this more accurate classification is to be preferred for its simplicity. Lichen is an eruption of full, solid, elevations, generally very small, sometimes slightly red, but most frequently of the colour of the skin, almost always agglomerated and accompanied by itching. It is not contagious. It has two well-marked forms; lichen simplex and lichen agrius.

1st. Lichen Simplex. This may be either acute or chronic. If acute, the papulæ, which are very small, and agglomerated in larger or smaller numbers, are slightly red and inflamed, accompanied by heat and itching; at the end of three or four days the redness diminishes, a slight desquamation takes place, and the disease terminates in about a fortnight, unless there are successive eruptions; it appears usually on the face and trunk. In the chronic form, which is the most common, the papulæ are of the colour of the skin, or of a light red colour; they are preceded by slight itching; the small elevations are readily felt by the fingers, giving the sensation of small hard bodies placed immediately beneath the upper skin. It is a very tedious complaint, remaining many weeks or months. The extremities and the dorsal surface of the hands are the situations on which it is found. It is seldom preceded by general symptoms. The papulæ are sometimes traversed by hairs (lichen pilaris, Willan); in individuals weakened by misery and privations they are livid (lichen lividus), but this is very rare; instead of being irregularly scattered the papulæ sometimes form regular circles, augmenting at their circumference by new eruptions, whilst the centre heals (lichen circumscriptus). M. Biett has described another species, the lichen gyratus, from the regular and twisted form produced by the groups of papulæ, which may be confounded with psoriasis gyrata. There are also two important varieties, the lichen urticatus and lichen strophulus, which includes the varieties of strophulus of Willan; both these forms are so well described by Bateman, that it would be superfluous to go into any detail here.

2d. Lichen Agrius. This may exist spontaneously, or succeed to simplex. Numerous small elevated papulæ, very red and inflamed, are formed on an erythematous surface; the surrounding skin is inflamed and painful; instead of these symptoms diminishing on the fourth day, as in lichen simplex, the inflammation augments, the papulæ increase in size, their summit ulcerates, a sero-purulent liquid is formed, which produces small yellowish elevated scabs, not rough but soft, and but slightly adherent. Sometimes the inflammation ceases, and the eruption terminates by desquamation in about a fortnight. The itching is so troublesome that the disease is aggravated by the constant scratching which it induces. It sometimes becomes chronic, and the scabs are replaced by a branny exfoliation, accompanied by a chronic thickening of the skin; this state may last many months.

Lichen affects all ages; it commences usually in the Spring and Summer; arising frequently from a disturbed state of the general health, or from direct irritating applications.

*Diagnosis.* Often difficult. Lichen simplex may be confounded with eczema, scabies, and prurigo. The papulæ of prurigo are situated, similarly to those of lichen, on the extended surfaces of the limbs, but they are larger and flatter; the summit of many is covered by a black scab formed of dried blood produced by the scratching of the patient: the pruritus in prurigo is more burning and excessive than in lichen, which is frequently very slight.

Lichen circumscriptus may be mistaken for herpes circinnatus, but the circles of herpes are more inflamed, and those of lichen, although more elevated, are often of the colour of the skin. Papulæ can be found in the centre of the patches of lichen circumscriptus, whilst the skin in the centre of herpes circinnatus is perfectly healthy. When the vesicles of herpes cannot be seen, numerous small round points surrounded by a narrow white border, which formed the base of each vesicle, are found.

Lichen urticatus may be confounded with erythema papulatum, but the patches of this eruption are larger, less red and elevated, and never are accompanied with the severe itching with which the other is attended; it differs from syphilitic lichen from the absence of the coppery colour: this eruption is slower in its progress, and is never attended with pruritus. The diagnosis between lichen simplex and eczema and scabies has been given in the descriptions of those eruptions. Lichen agrius may be confounded with acute and chronic eczema, impetigo and psoriasis. But the thin, soft, and slightly adherent scabs are very different to those of impetigo; and besides, papulæ may be found around the margin of the eruption. The scales of psoriasis are larger than the branny exfoliations of the chronic form of lichen agrius. The differences between this and eczema have before been pointed out.

*Treatment.* The acute form of lichen simplex requires merely diluent drinks, tepid or cold baths; when it is chronic, slight laxatives, alkaline or sulphur baths, and sometimes frictions, with ointments composed of calomel, ʒss. camphor, gr. xij. lard, ℥j. or of 12 grs. to a scruple of proto-ioduret of mercury with an ounce of simple ointment. The antiphlogistic treatment, with general or local bleedings, emollient poultices, and tepid baths, is to be employed in the first stage of lichen agrius, if attended with much inflammation, also purgatives of calomel and castor oil. If the eruption is obstinate, arsenical preparations, such as Fowler's solution, five drops of which is to be taken daily, and gradually increased to 25 or 30 drops, watching its effects on the bowels with great caution, and suspending it when at all necessary; the local applications are similar to those recommended in the chronic lichen simplex.

*Prurigo.* Both the divisions of this troublesome complaint, and the treatment, as laid down by Bateman, are followed by Biett. In prurigo podicis the cinnibar fumigations have been found very beneficial, but as they diminish the strength of the patient when the whole body is subjected to their influence, M. Biett has introduced into the Hôpital St. Louis an apparatus by which the fumes are applied only to the part affected.

### Squamæ.

The classification of Willan is followed, no alteration having been made in the number of genera.

*Lepra.* M. Biett recognises but one form of lepra, the lepra vulgaris of Willan; the lepra alphoides differs from it only in the lighter colour of the patches, owing to the eruption affecting the young and feeble; lepra nigricans is a scaly syphilitic eruption. As the description of lepra vulgaris coincides exactly with that given by Bateman, we shall pass to its diagnosis. Porrigo scutulata at the commencement, or after the incrustations have fallen off, leaving a red annular surface, may, for a short period, be mistaken for lepra vulgaris affecting the scalp; but, by watching its progress, the eruption of favi, the nature of the incrustations, the destruction of the bulbs of the hair, and the contagious character of porrigo, will sufficiently distinguish the two affections. The tubercular syphilitic eruption, from the circular form which it sometimes assumes, may be mistaken for lepra; but, on examination, the circles will be found not to be continuous, but formed of isolated tubercles, having distinct intervals left between them; these tubercles are shining, elevated, not covered with scales, or at least, in some few cases, but partially covered with extremely small and hard lamellæ; besides the coppery or violet tint, the cicatrices which are generally found in the neighbourhood of the eruption, and the concomitant general symptoms, render the diagnosis more easy. Lepra vulgaris is usually distinguished from psoriasis with much facility by the irregular patches of the latter eruption; but the circular spots of psoriasis guttata may be confounded with lepra, particularly when this eruption is declining; on careful examination, the spots of psoriasis guttata will be found to be less regular in their shape, always smaller, and presenting no depression in the centre, free from scales. When, during an extensive eruption of lepra, the circles unite and become confounded, they may simulate the irregular patches of psoriasis, but parts of distinct circles will be found around the circumference.

*Treatment.* M. Biett has found the three following methods most successful in this frequently intractable complaint. 1. By purgatives, according to the treatment of Hamilton. This is used when the eruption is recent and not extensive; and particularly when it affects children. The most useful purgative is four grains of calomel, with the same quantity of powdered jalap, administered every morning; it is not unusual to obtain a cure by this method in two months, and it rarely salivates. In some cases, a daily dose of ʒij. or ℥ss. of sulphate of magnesia or soda, given in a weak bitter infusion, is sufficient: at other times more drastic purgatives, as aloes, colocynth, and gamboge, according to the strength of the patient and state of the eruption. 2. By tincture of cantharides. The effect of this is often surprising when given in cases which have resisted purgatives where the eruption is of great extent and the constitution feeble. The patient should be restricted to a low diet, and from three to five drops should be given in a teaspoonful of water every morning; this dose should be gradually increased, adding to it five drops every six or eight days, and watching with care its effect on the digestive and genito-urinary organs; if it produces nausea, heat in the epigastrium, ardor urinæ, erections, &c. (which are seldom caused by it), its use must not be persevered in until these symptoms decline; in this cautious manner, the daily dose may be gradually increased to twenty-five or thirty drops; frequently, and particularly in females, the eruption is wholly cured by this medicine in 45 or 50 days. A case of lepra at the Hôpital St. Louis was cured in one month by this tincture, which had existed for 18 years. 3. By arsenical preparations. Where lepra has existed many years, where it covers great part of the body, and produces a chronic thickening of the skin, arsenic is of signal service. Three drops of Fowler's solution every morning, gradually augmented to twenty or twenty-five, beyond which quantity it is improper to administer it, or twenty minims of Pearson's solution, increased gradually to a drachm, are the most convenient forms for administering arsenic. Great care must be paid to its action on the digestive organs. The most useful topical application is the ointment of ioduret of sulphur, particularly in cases in which energetic internal remedies are prohibited by the weak state of the patient. Sulphur-baths and vapour-baths very beneficially assist the action of the other remedies.

*Psoriasis.* The four varieties of this eruption and their descriptions, agree precisely with those of Willan and Bateman. The treatment to be employed is that which has been recommended in lepra. In psoriasis inveterata, M. Biett has administered with success a preparation of arsenic, known in France by the name "pilules Asiatiques;" this consists of fifty-five grains of protoxyde of arsenic, mixed with nine drachms of pepper, and made up into eight hundred pills, each pill contains about 1-30th of a grain; one pill must be commenced with daily, and the dose increased to two, but never more. He has also found very minute doses of arseniate of ammonia, such as the 30th of a grain given daily, remove, in a short time, this inveterate eruption. In psoriasis palmaria, as well as in other local varieties, much advantage has been derived from frictions on the diseased surface, which has been previously softened by warm emollient baths, with ointments containing the proto-ioduret of mercury, or the ioduret of sulphur, in the same quantities before prescribed.

*Pityriasis.* The descriptions and treatment are similar to those of Willan and Bateman, with the exception of pityriasis versicolor, which our authors call an ephelis; Bateman, although he has followed Willan in his arrangement of varieties, considers that these two eruptions differ but little, and that pityriasis versicolor frequently degenerates into an ephelis. The desquamation following chronic eczema and lichen may be confounded with pityriasis; but the vesicles of the first or their remains, and the papulæ and thickened skin of the second eruption, as well as the constant renewed formation of the scales of pityriasis, which never happens in the others, sufficiently distinguish them.

*Icthyosis.* Both M. Biett and M. Alibert consider that this eruption is most frequently congenital and hereditary; this is not mentioned by Bateman; he merely states "that it often commences in childhood, and often in early infancy." When congenital, the skin of the newly-born infant, instead of presenting a smooth, polished surface, is dull, thick, and furrowed, or remarkably dry, and covered by small scales of a grey colour, formed by the exfoliated epidermis; as the infant becomes older, the characters of the eruption are more marked; these are admirably described by Bateman. M. Biett considers that congenital icthyosis is a disease incurable by medicine; palliatives may be prescribed, such as mucilaginous baths and lotions, vapour-baths, &c. to render the skin less dry and hard; indeed, in the other forms of icthyosis, these are the only means which have been found serviceable at the Hôpital St. Louis. In the post-mortem examinations of those who have been affected with icthyosis, no organic disease has been discovered which could be connected with the eruption; indeed the patients usually enjoy good general health.

### Tuberculæ.

M. Biett admits only three classes—elephantiasis of the Greeks, framboesia, and molluscum.

*Elephantiasis of the Greeks.* The term "elephantiasis" has been indiscriminately applied, by many modern writers, to a tubercular eruption and to a chronic enlargement of the cellular tissue of the leg, which, from its being an endemic complaint in an island of the West Indies, has been called "the Barbadoes leg." Dr. Bateman, in his learned history of elephantiasis, has endeavoured to reconcile the descriptions of the ancients with those of the more modern authors, and has decided that the Greeks applied the term ελεφας and ελεφαντιασις to a tubercular eruption (a very accurate drawing of which he has given in his quarto volume of plates), and that the Arabs have described by the same name a chronic enlargement of the skin and cellular tissue of the legs. Dr. J. Mason Good has stated that "dal fil" is the common name for the swelled leg, in the present day, among the Arabians, who sometimes contract it to "fil" alone, literally "elephas." The confusion was still more increased, by the translators of the works of the Arabian physicians into Latin naming elephantiasis the "Arabian leprosy." Elephantiasis of the Greeks is placed among tuberculæ, that of the Arabs in the anomalous orders. The description which Dr. Bateman has given of this tubercular eruption is, as he candidly acknowledges, drawn from books, as he had seen but four cases. The following description is drawn up from the lectures of M. Biett, who has had more opportunities of observing this eruption. The face and limbs are most frequently the seat of the eruption. The tubercles are preceded by dark spots on the skin of whites, of yellowish or reddish on blacks; they are at first elevated, irregular, red or livid, and soft, from the size of a pea to that of a nut; sometimes indolent, at other times acutely sensible; the intervening skin is hypertrophied, causing great deformity. The eruption may remain stationary for a long time or make rapid progress, the whole face being covered by knotty tumours, divided by deep furrows; the nostrils become dilated, the nose, lips, and ears monstrously enlarged; the eyebrows and ciliæ fall out. The limbs are oily and shining, covered with enormous flattened tubercles; the subjacent cellular tissue is tumefied; the sensibility, which was in the first instance excessive, gradually diminishes until all feeling is lost, as well as the voice and smell, whilst the senses of vision and touch are singularly deteriorated; the moral energies are similarly weakened and depressed. The "libido inexplebilis" which is said to accompany this disease, has not been observed in the cases admitted into the Hôpital St. Louis. In the most severe cases the tubercles ulcerate, the foul ulcers pour out a sanies which forms adherent blackish scabs. The mucous membrane lining the mouth and covering the tongue, as well as that of the conjunctiva, is covered with tubercles. On examinations after death, the gastro-intestinal mucous membrane is almost always discovered to be in a diseased state, and the immediate cause of death is, in the greater number of cases, extensive ulceration of the ileum, the ileo-cæcal valve, and the colon. The tubercles on the membrane lining the mouth are found ulcerated, and they are sometimes traced as far as the ventricles of the larynx. Tubercles (that is pulmonary tubercles, as described by Laennec, &c.) are found in the lungs and mesentery; Alibert and Ruette have described the bones, in some cases, as soft and spongy. Elephantiasis is often hereditary, but not invariably so. M. Biett attended a West-Indian lady who gave birth to several perfectly healthy children during the progress of this disease. Almost all the individuals met with in France, affected with elephantiasis, are natives of the colonies.

*Prognosis.* It is always a serious disease; usually a fatal one. The patients, morose, sad, debilitated, discouraged, and totally deprived of the greater number of their senses, are destroyed by a slow fever; or, from an extension of the eruption along the mucous surfaces, death is produced by the most intense chronic gastro-enteritis. In some rare cases, where the eruption is recent and of small extent, the tubercles are gradually absorbed.

*Treatment.* If the individual affected with tubercular elephantiasis applies for relief at the commencement of the disease, dry frictions, liniments, and particularly blisters, are to be employed, to increase the vitality of the skin. When the eruption is more advanced, but still circumscribed, as, for example, to a part of the ear, frictions of an ointment composed of hydriodate of potash, ℈j., lard, ℥j., or douche vapour-baths, directed on the part for 15 or 20 minutes daily. When more extensive, and the general health permits it, sudorifics, tincture of cantharides, and arsenical preparations, in the doses recommended in lepra, may be tried. When the mucous membranes are attacked, soft mucilaginous drinks, low diet, opiates, &c. are to be substituted. It is always advantageous to change the climate.

*Frambœsia—the Yaws.* This tubercular disease, which is indigenous in Africa, and very common in the West Indies, has been met with more than once by M. Biett in France. It is well described by Bateman.

*Molluscum.* Nothing has been added to the description of this disease given by Bateman; M. Biett has never seen the contagious variety. A case confirming the two cases of contagious molluscum, given by Bateman, was communicated to our authors by Dr. Carswell (now a Professor at the London University), which fell under his inspection in Edinburgh.

We must make the consideration of maculæ, and the anomalous orders, the subject of another article.

